# Drying Spring Accelerates Transitions Toward Pyrogenic Vegetation in Eastern Boreal North America

**DOI:** 10.1111/ele.70166

**Published:** 2025-06-26

**Authors:** A. A. Ali, D. M. Gaboriau, J. A. Lesven, M. P. Girardin, C. C. Remy, D. Arseneault, G. de Lafontaine, V. Danneyrolles, H. Asselin, F. Gennaretti, E. Boucher, P. Grondin, M. Garneau, G. Magnan, B. Fréchette, S. Gauthier, Y. Bergeron

**Affiliations:** ^1^ Institut Des Sciences de L'évolution Université de Montpellier (UMR 5554) Montpellier France; ^2^ Institut de Recherche Sur les Forêts Université du Québec en Abitibi‐Témiscamingue Rouyn‐Noranda Quebec Canada; ^3^ Laboratoire Chrono‐Environnement, UMR 6249 CNRS Université Marie et Louis Pasteur Besançon Cedex France; ^4^ Natural Resources Canada Canadian Forest Service, Laurentian Forestry Centre Quebec City Quebec Canada; ^5^ Département de Biologie, Chimie et Géographie Université du Québec à Rimouski Rimouski Quebec Canada; ^6^ Département Des Sciences Fondamentales Université du Québec à Chicoutimi Chicoutimi Quebec Canada; ^7^ École D'études Autochtones Université du Québec en Abitibi‐Témiscamingue Rouyn‐Noranda Quebec Canada; ^8^ Groupe de Recherche en Écologie de la MRC‐Abitibi (GREMA), Institut de Recherche Sur les Forêts Université du Québec en Abitibi‐Témiscamingue Amos Quebec Canada; ^9^ Ministère des Ressources naturelles et des Forêts Direction de la recherche forestière Québec Quebec Canada; ^10^ Centre de Recherche Geotop Research Centre Université du Québec à Montréal (UQAM) Montreal Quebec Canada

**Keywords:** boreal forest, climate change, fire regime, paleoecology, *Picea mariana*, *Pinus banksiana*

## Abstract

The ongoing climate change increases vegetation flammability in the boreal forests of eastern North America, leading to more intense and severe wildfires. Using palaeoecological data—including charcoal, pollen, chironomids and testate amoebae—and climate model simulations of vapour pressure deficit (VPD) and available soil water (ASW), we analysed fire dynamics over the past 8000 years in boreal eastern North America. Over the last 4000 years, and particularly in the last 250 years, increasing spring drought has led to fewer, but more severe fires. This shift in the fire regime has favoured the spread of fire‐adapted conifer species, particularly jack pine ( *Pinus banksiana* ), across the landscape. We infer that the predicted increase in VPD and decrease in ASW triggered by climate change will alter the fire regime and amplify the transition toward more pyrogenic vegetation within the boreal forest of eastern North America, with ecological and socio‐economic consequences.

## Graphical abstract

Over the past 8000 years, spring climatic conditions, particularly drought, have been the primary driver of wildfire dynamics in eastern boreal North America. Increasing spring dryness over the last 4000 years has favoured larger and more severe fires than before, promoting the spread of fire‐adapted species like jack pine across the landscape.

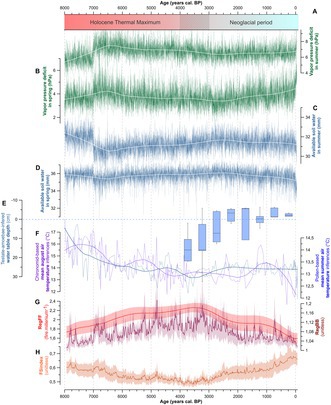

## 1 Introduction

The circumboreal biome plays a key role in regulating global climate through its contribution to the carbon cycle (Girona et al. 2023; Morineau et al. 2023; Spawn et al. 2020; Thom and Seidl 2016). Although boreal forests are generally characterised by long, cold winters and short, warm summers (Bonan and Shugart 1989), these ecosystems are currently experiencing amplified warming (Rantanen et al. 2022) and are increasingly affected by larger, more frequent and/or more severe fire events (Venäläinen et al. 2020; Wang et al. 2017). A critical climate forcing affecting fire regimes is the atmospheric vapour pressure deficit (hereafter VPD), which reflects the disparity between the moisture content in the air and its moisture‐holding capacity at a given temperature. Rising temperatures generally increase the VPD (Will et al. 2013), triggering an atmospheric evaporative demand that forces the extraction of moisture from plants, organic matter and soils, which promotes vegetation flammability and fire susceptibility (Clarke et al. 2022; Jain et al. 2022; Parks and Abatzoglou 2020; Wang et al. 2023). Thus, while climate warming can enhance forest productivity (D'Orangeville et al. 2018; Lesven et al. 2024), it also induces drier conditions, significantly escalating fire risk (Chaste et al. 2019; Gaboriau et al. 2023).

Wildfires in North America were exceptionally severe in 2023, particularly in Canada, where over 15 million hectares of forest were consumed, resulting in more than 2.1 Gt of carbon emissions (Boulanger et al. 2025; Byrne et al. 2024; Soulie et al. 2024). As a result, 2023 became the year with the largest area burned by wildfires on record in Canada, based on national inventories dating back to the 1960s (Jain et al. 2024). The occurrence of such extreme wildfire years, driven by unprecedented climate conditions, could not only affect forest structure and biodiversity but also cause health and safety issues for local populations, thus raising a critical question: are we witnessing a disruption of historical fire regimes, potentially signalling a new era of intensified fire activity? One way to address this question is to consider the 2023 fire season in the light of historical fire records dating back several centuries/millennia. In this broader context, paleoecological and retrospective studies are used to understand past climate change and assess its consequences on forest dynamics and disturbance regimes. Paleoecological data from lake and peat sediments, including charcoal records, pollen assemblages and other proxies such as chironomid and testate amoebae, can be jointly analysed to provide compelling reconstructions of long‐term changes in fire regimes, vegetation dynamics and climate conditions (Girardin, Gaboriau, et al. 2024). Additionally, paleoclimatic simulations from global climate models (GCMs) provide complementary data about long‐term climate changes, such as relative humidity or VPD, in response to known variation in climate forcings. By integrating GCM outputs with paleoecological data, a mechanistic understanding of past climate dynamics and their effects on fire regimes and vegetation patterns can emerge (Hély et al. 2010). Such an integrated approach enhances our ability to incorporate modern‐day observations into a long‐term perspective and to project future fire activity and vegetation trajectories (Girardin et al. 2013).

**FIGURE 1:**
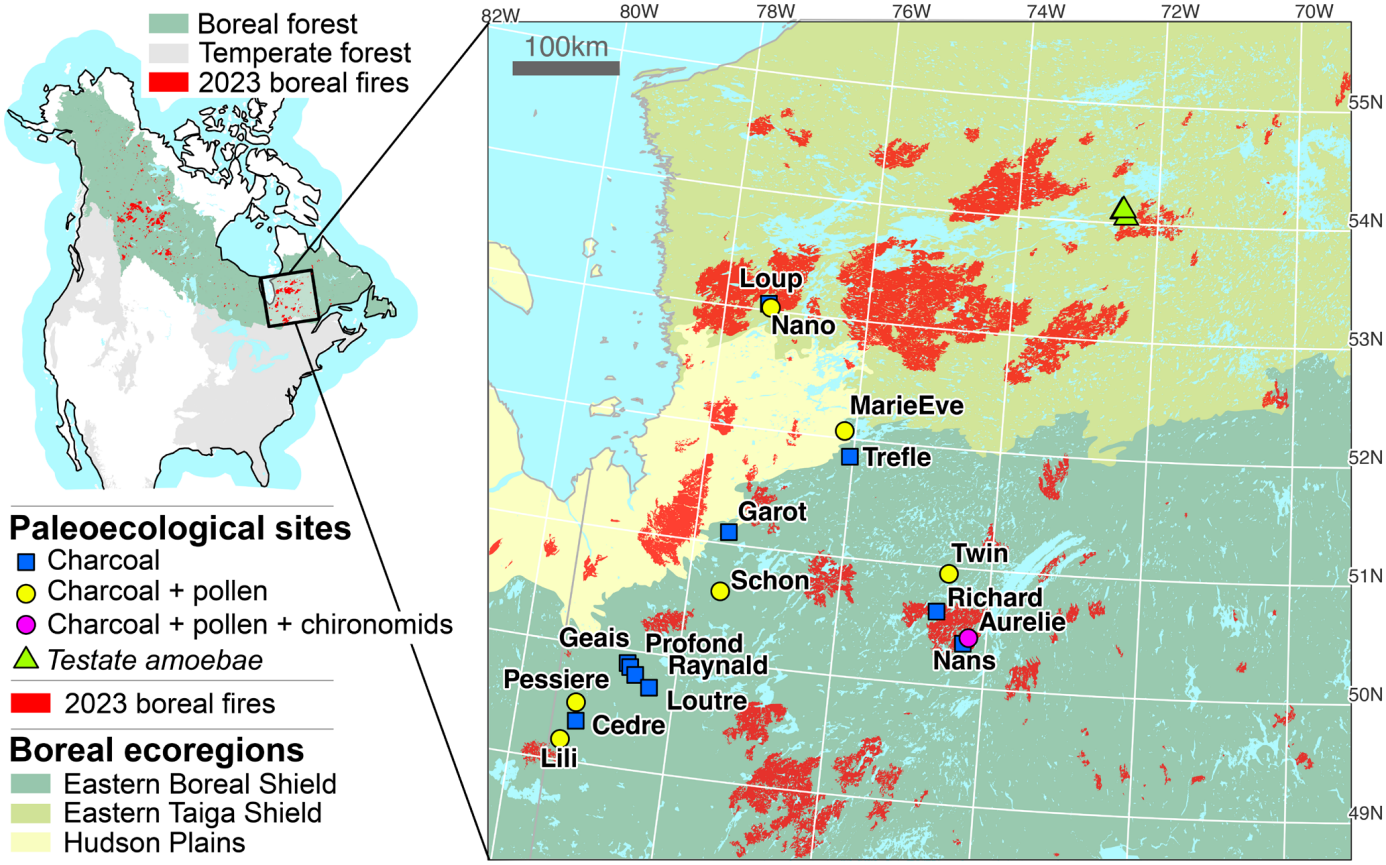
Location of the study area and proxies used in the study.

Taking advantage of a large corpus of paleofire reconstructions and climate simulations, this study reconstructed 8000 years of climate–fire–vegetation interactions in the coniferous boreal forest of eastern North America (Figure 1; Table S1). The study sites are located in eastern Canada within the Boreal Shield ecoregion (Olson et al. 2001), near the transition with the Eastern Taiga Shield ecoregion (Figure 1). This area has been largely affected by fires during the 2023 season and in recent decades (Figure 1). The dominant conifer species currently established in the area are black spruce ( *Picea mariana* (Mill.) B.S.P.) and jack pine ( *Pinus banksiana* Lamb.). We compiled charcoal, pollen and chironomid datasets from 17 lake sediment deposits, along with testate amoebae assemblages from peat sediments (Figure 1; Table S1). Additionally, climate simulations and a bioclimatic model were used to characterise historical variations in VPD and available soil water (hereafter ASW). Building on current understanding of boreal fire–climate interactions, we hypothesised that periods of elevated spring and summer VPD during the Holocene, especially those coinciding with limited ASW, would correspond with increased fire frequency and biomass burning. We also expected that long‐term shifts in climate seasonality would influence not only fire frequency but also individual fire size and subsequent vegetation recovery. These hypotheses provide a framework to evaluate how fire‐climate‐vegetation dynamics have evolved over millennia and to determine whether recent trends signal a departure from, or a return to, past fire‐prone conditions in eastern boreal North America.

## 2 Material and Methods

### 2.1 Chronological Setting and Age‐Depth Models

Accelerated mass spectrometry (AMS) radiocarbon dating (^14^C) was used to establish the chronology of sedimentary cores from the 17 sites (Table S1). Radiocarbon dating was done on terrestrial plant macroremains (i.e., needles, seeds) or bulk organic sediments. The ^14^C dates were calibrated in years before present (BP) using age‐depth models computed using Bayesian age‐modelling with the ‘rbacon’ v.3.1.1. R package (Blaauw et al. 2021). We used the IntCal20 Northern Hemisphere Radiocarbon Age Calibration Curve for terrestrial material (Reimer et al. 2020) and generated confidence intervals (CI) around the fit based on the probability distribution of each date. Ages were then interpolated at contiguous 1‐cm depth intervals.

### 2.2 Wildfire History Reconstructions

#### 2.2.1 Reconstruction of Past Biomass Burning

We used charcoal particles sequestered in lake sediments to reconstruct wildfire history (Data S1). Past biomass burning was inferred from total charcoal influx (hereafter CHAR; mm^2^ cm^−2^ year^−1^) using sediment accumulation rates obtained from the age‐depth models (Clark 1990; Cwynar 1987; Larsen and MacDonald 1998; Power et al. 2008; Figure S1). A composite record of regional biomass burning (RegBB) was constructed by pooling Z‐score–transformed individual CHAR series (Ali et al. 2012; Marlon et al. 2008, 2012; Power et al. 2008; Data S1). This method minimised biases related to taphonomic processes and changes in sedimentation accumulation rates.

#### 2.2.2 Reconstruction of Past Fire Events and Frequency

We used CharAnalysis v.1.1 (https://github.com/phiguera/CharAnalysis; Higuera 2009) to detect past fire events for each interpolated individual CHAR series (Figure S2). Each CHAR peak exceeding a given threshold was assumed to be a fire event representing one or several fires occurring within the source area and within the median sampling resolution of each sequence (Data S1). Then, the frequencies of fire events at each site were averaged into a regional fire frequency record (hereafter RegFF; fire.year^1^) by adjusting the fire frequency values to the changing number of lake samples through time (Ali et al. 2012).

#### 2.2.3 Fire Size/Severity Index

The FS index was calculated as the ratio of RegBB to RegFF over time (Ali et al. 2012; Kelly et al. 2013, Data S1). This ratio relies on the premise that individual fire size/severity is related to the temporal trajectory of mean biomass burned per fire, i.e., the loss of biomass (RegBB) modulated by the number of fires over time (RegFF).

### 2.3 Vegetation History Reconstructions

Pollen records from six lakes were used to decipher how regional vegetation dynamics interacted with fire and climate during the Holocene (Figure 1, Data S1, Table S1). For each lake, 1‐cm^3^ subsamples were extracted at intervals ranging from 2 to 4 cm in the sediment cores, and then chemically treated following standard procedures (Faegri et al. 1989) in preparation for pollen identification. Pollen and spores were counted and identified at ×400–1000 magnification using modern pollen collection at the Centre for Northern Studies (Université Laval, QC, Canada) and dichotomous keys (McAndrews 1973; Moore et al. 1991; Richard 1970). A minimum of 300 to 500 pollen grains from terrestrial vascular plants were included for every pollen spectrum to quantify percentages of the terrestrial vascular plant pollen sum. The percentages for each species at each site were interpolated over a 10‐year time step to calculate the regional mean and standard error.

### 2.4 Climate Simulations

Climate simulations relied on spatially comprehensive datasets of monthly mean daily minimum and maximum temperatures, and precipitation spanning the Holocene, sourced from TraCE‐21 ka (‐II) climate simulation (Clarke et al. 2022). The simulation integrates the Earth's orbital variations, greenhouse gas forcings and improved modelling of Atlantic Ocean circulation dynamics influenced by melting polar ice (https://trace‐21k.nelson.wisc.edu/Data.html). We focused on our study zone between 80°W and 70°W and 48°N to 54°N. Average data for this area were extracted for a period covering 8.2 ky BP to ad 1990. To correct for biases in model simulation, we adjusted the means of the simulation data to match the observed mean for 1950–1990 obtained from the Climate Research Unit TS 4.07 and extracted from the KNMI Climate Explorer (https://climexp.knmi.nl/).

We used the vegetation model StandLEAP (Girardin et al. 2011, 2016, Data S1), based on the Physiological Principle Predicting Growth (3PG) model, to generate past fluctuations in VPD (hPa) and ASW (mm) for the region (Landsberg and Waring 1997). For this work, the StandLEAP model was initialised and ran using the 8200 years of TraCE‐21 ka (‐II) monthly climate data, atmospheric CO_2_ concentration and plot information from 284 locations. Plot information included forest composition (% cover), above‐ground biomass (Mg/ha), tree density (stems/ha), stand age (years), mineral soil texture (% sand, clay, silt), aspect, and slope (°) derived from a sampling programme of 400 m^2^ circular sample plots (Girardin et al. 2012). Active soil depth, which represents the zone of root water uptake, is prescribed in the model to range between 200 and 1400 mm. To maintain consistency, forest attributes such as biomass and stem densities were assumed constant across all 8200 simulation years (Girardin et al. 2016), ensuring that carbon flux quantities reflect direct climatic influences on RUE and plant respiration.

### 2.5 Climate Proxy Records

#### 2.5.1 Testate Amoebae

Water table depth (WTD) reconstructions of the study area were realised using testate amoebae from five peatlands (Table S1) of the Laforge region in north‐central Quebec (Van Bellen et al. 2013). A transfer function built from a modern testate amoebae training set covering sites from Quebec (between 45°N and 55°N) was used to infer water table levels (Lamarre et al. 2013). The transfer function was developed from 206 surface peat samples collected from 18 peatlands across boreal Quebec (Lamarre et al. 2013). We used a weighted average model with tolerance downweighing and classical deshrinking. Water table levels were expressed as depths relative to the peatland surface, i.e., negative values indicate standing water. In peatlands, although WTD variations can be driven by internal processes such as vertical peat growth, long‐term fluctuations in water table levels mostly reflect changes in the atmospheric water balance (precipitation minus evapotranspiration) and particularly the summer moisture deficit (Charman et al. 2009).

#### 2.5.2 Chironomids

To infer mean August air temperatures, we used previously published chironomid data from Aurélie lake (Bajolle et al. 2018). Temperatures were derived from chironomid assemblages using the eastern Canadian transfer function of Larocque (2008), comprising 79 chironomid taxa from 75 lakes in eastern Canada. Mean August air temperatures were inferred using a weighted average–partial least squares model with two components. The 999‐bootstrap transfer function gave a correlation coefficient (*r* ^2^ boot) of 0.85, a maximum bias of 3.05°C, and a root mean square error of prediction (RMSE) of 1.67°C (Bajolle et al. 2018). For this study, only samples younger than 8.0 ky BP were considered and smoothed using a loess regression (span = 0.3).

#### 2.5.3 Pollen

The modern training set was derived from the North American Pollen Database (Whitmore et al. 2005), restricted to eastern North America and Greenland (Fréchette et al. 2021), and comprised 56 pollen taxa from 2418 sites, each with a pollen sum exceeding 100 grains. Fossil pollen from Aurélie, Marie‐Eve, Nano, Pessière, Schön, and Twin was converted to mean summer temperatures using the modern analogue technique (MAT), based on the squared Hellinger distance (Legendre and Gallagher 2001). The optimal number of analogues (*k* = 4) was determined by ranking five performance metrics (root mean square error, *R* ^2^, skill score, mean and maximum bias), and mean summer air temperature estimated as the weighted average of the climatic values of the analogues. Temperature estimates were then linearly interpolated, averaged across the six sites and smoothed using a loess regression (span = 0.3).

### 2.6 Statistical Analyses

The significance of changes in RegFF, RegBB and FSindex was evaluated using a bootstrap procedure applied to sites with 999 iterations (BCI; 90%). Changes in these fire metrics were considered significant if the 90% BCI did not overlap between two periods. Relationships among mean regional fire metrics (RegFF, RegBB, RegFS), climate and relative pollen abundances, were examined using binned correlation procedures (Polanco‐Martínez et al. 2018). The approach enables correlation assessments among time‐series datasets with dissimilar temporal characteristics, making it particularly useful for analysing paleoenvironmental data with varying sampling frequencies and temporal resolutions. We used the Spearman rho coefficient, with 95% confidence interval, for quantification of the magnitude of relationships; the bin‐widths were estimated for each pairwise comparison considering the persistence (memory) estimated for each unevenly spaced time series. The results are visually depicted using a heatmap diagram. The investigation period ranged from 8000 to 0 ky BP. The analysis was conducted using the R package BINCOR (Polanco‐Martínez et al. 2018).

A similar analysis was repeated using the simulated StandLEAP and fire data over the instrumental 1950–2022 period. The Spearman correlation coefficient was used to quantify the strength of relationships, considering autocorrelation persistence (memory) estimated for each time series (PearsonT3 software; Ólafsdóttir and Mudelsee 2014). We also verified the magnitude of climatic effects by testing for differences in medians of annual area burned, mean fire size and number of fires during the 10 years of lowest and highest VPD and ASW conditions. Statistical significance at the 5% level for these differences in medians was assessed using the accelerated bootstrap technique implemented in the 2SAMPLES software (Mudelsee and Alkio 2007).

## 3 Results

### 3.1 Fire‐Vegetation Nexus

**FIGURE 2:**
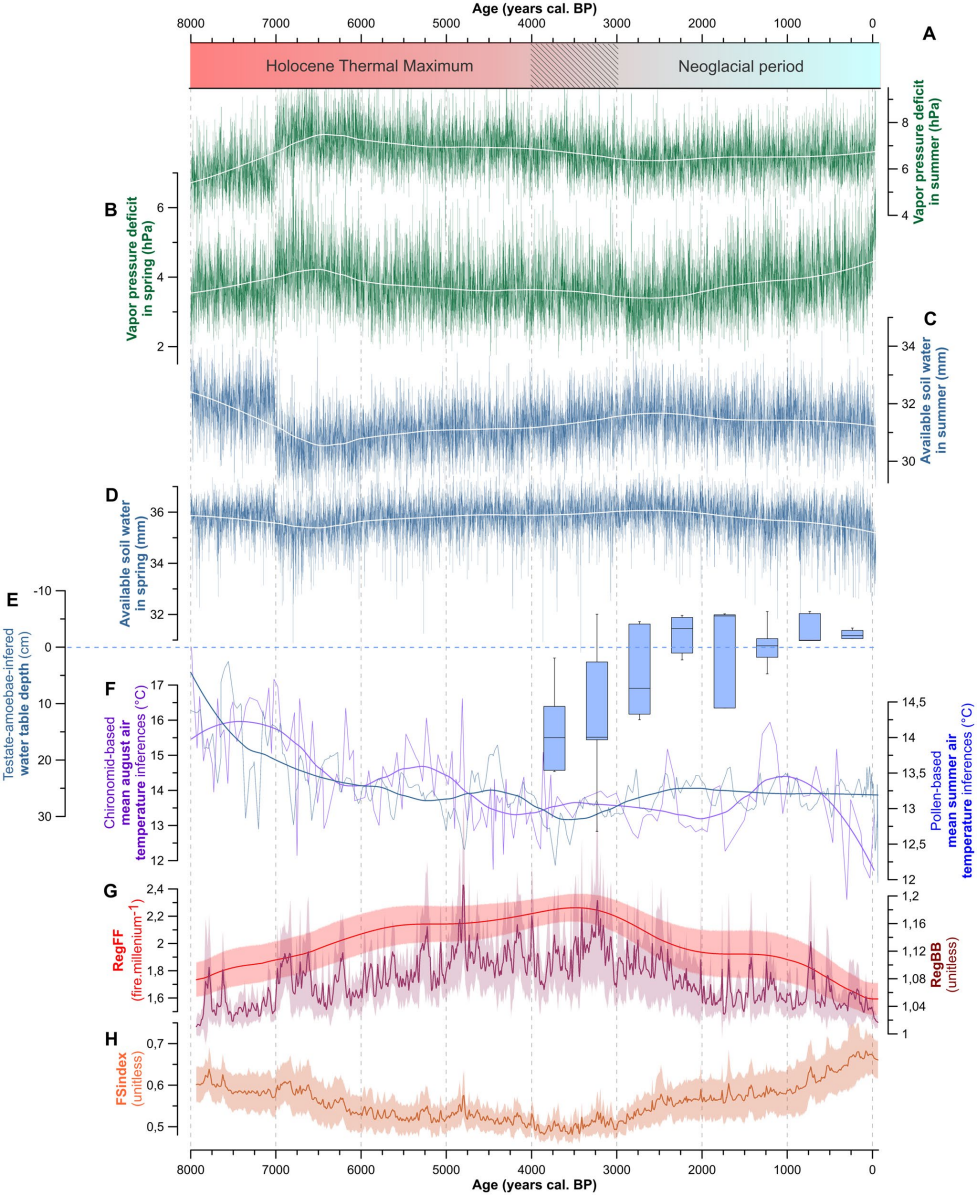
Regional fire and climate history during the Holocene. (A–D) Simulated average spring and summer vapour pressure deficit (VPD, in hPa) and available soil water (ASW, mm) generated from monthly temperature and precipitation outputs from TraCE‐21 ka‐II Holocene climate simulations. The white line represents a loess regression smoothing (span = 0.3). Increasing VPD denotes a drying atmosphere, whereas a lowering of ASW denotes depleting soil moisture content. (E) Water table depths pooled in 500‐year bins, representing medians, upper and lower quartiles and outliers. (F) Mean august air temperature reconstructed from chironomids (purple) and mean summer air temperatures inferred from pollen grains (blue). The thin curves represent the reconstructed values, and the thick curves the loess regression smoothing (span = 0.3). (G, H) Mean regional fire size and severity index (FSindex), biomass burned (RegBB) and fire frequency (RegFF), interpolated from charcoal accumulation rates, with 90% confidence intervals (CI). The hatched area on the y‐axis corresponds to the time during which the principal changes in fire regime vegetation and climate occurred.

**FIGURE 3:**
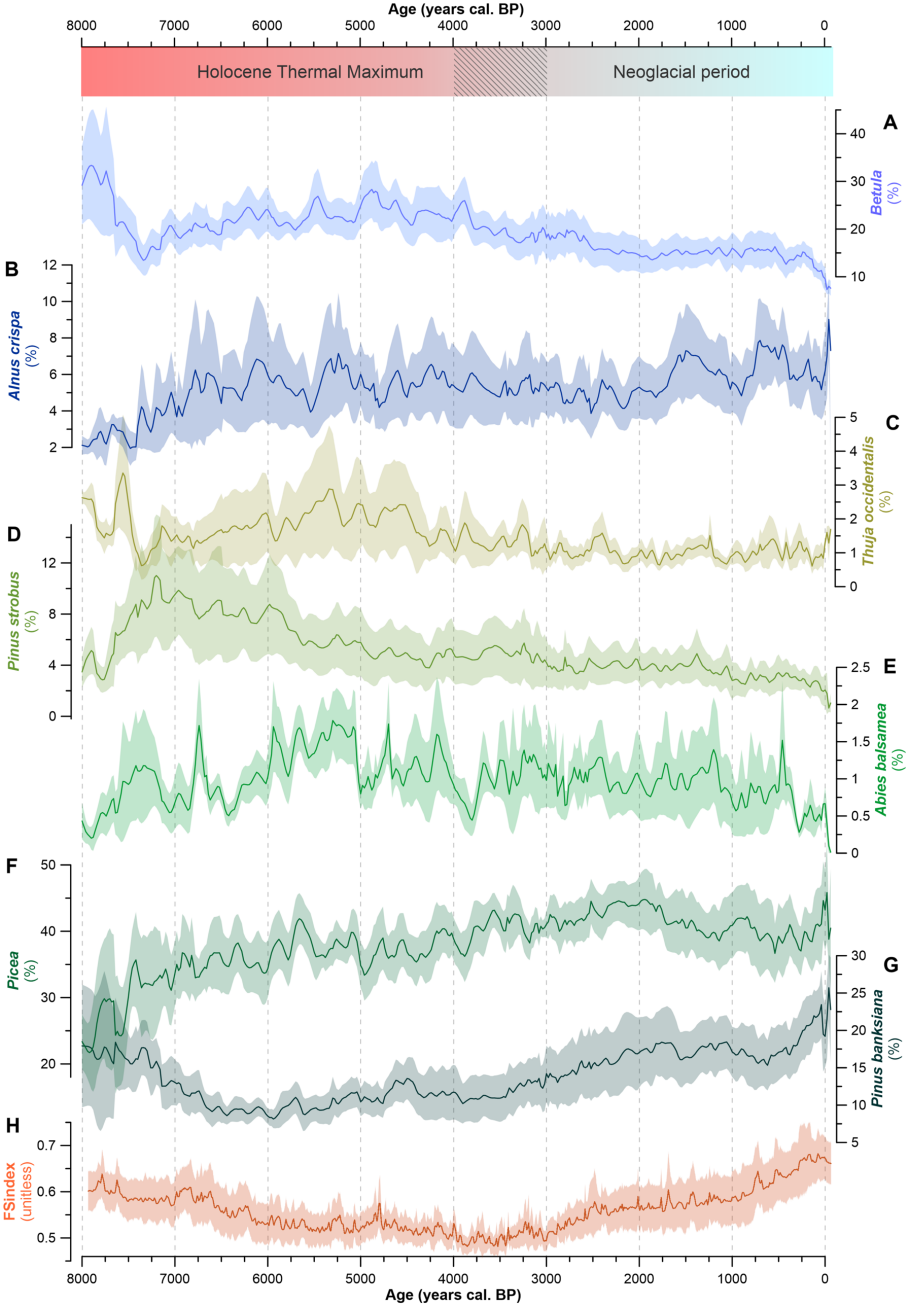
Regional vegetation changes alongside changes in mean regional fire‐size/severity. (A–G) Mean regional relative pollen abundances (%) with the standard error for the main taxa computed from lake sediment records. (H) Mean regional fire size and severity index (FSindex). The hatched area on the *y*‐axis corresponds to the time during which the principal changes in fire regime vegetation and climate occurred.

Composite charcoal records indicate a gradual increase in fire frequency from 8.0 to 4.0 ky BP, with high levels of biomass burning (Figure 2G). The mean fire size index (hereafter FS index) (Ali et al. 2012; Remy et al. 2017) suggests a concurrent steady reduction of individual fire extents during this period (Figure 2H). Pollen data indicate that the vegetation was mainly composed of black spruce (15%–35% relative abundance, Figure 3F), jack pine (10%–15%, Figure 3G), birch (*Betula* sp., 20%–40%, Figure 3A), green alder ( *Alnus alnobetula* ssp. *crispa* (Aiton) Turrill, 4%–6%, Figure 3B), balsam fir ( *Abies balsamea* , 0.5%–2.0%, Figure 3E), eastern white cedar ( *Thuja occidentalis* L., 1%–4%, Figure 3C) and white pine ( *Pinus strobus* L., 4%–10%, Figure 3D). Relatively frequent but small fire events allowed both fire‐sensitive (balsam fir and eastern white cedar) and fire‐prone (jack pine and black spruce) conifer species to coexist within the Boreal Shield ecoregion. Between 8.0 and 4.0 ky BP, the regional vegetation likely resembled the modern mixedwood forests found south of the study area (Fréchette et al. 2018) and was modulated by more frequent but smaller fires compared to the northern boreal forest (Bergeron et al. 2004). As balsam fir is a poor pollen producer, its 2% relative abundance strongly suggests past dominance (Fréchette et al. 2018).

**FIGURE 4:**
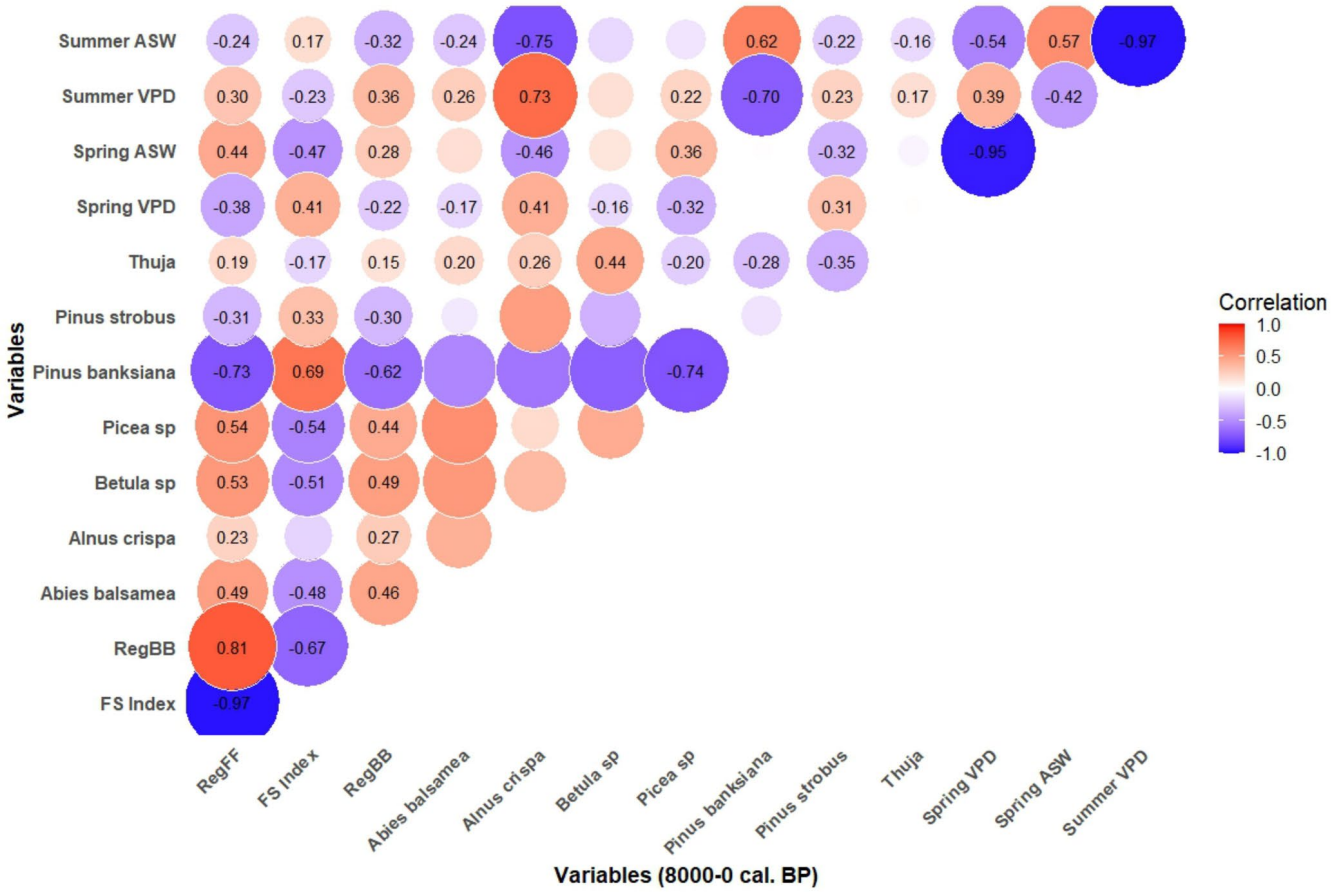
Relationships between Holocene (8000–0 cal. BP) seasonal dryness and fire regime components. The Spearman correlation coefficient was utilised to quantify the strength of relationships, accounting for the autocorrelation of each time series. Results are visually represented using a heatmap diagram, illustrating by labels significant pairwise correlations among the variables at a 95% confidence level. Circles denote variable comparisons, with red indicating positive correlations and blue representing negative correlations. The size and colour gradient of the circles correspond to the magnitude of the correlation coefficients.

An increase in individual fire size after 4.0 ky BP (Figure 2H), peaking during the last 250 years, coincided with the growing dominance of fire‐prone species, including black spruce (35%–45%) and especially jack pine (10%–25%). Over the last 8000 years, jack pine abundance was positively correlated with fire size (Figure 4; Spearman's rho = 0.69; *p* < 0.0001). The spatial distribution and regeneration of this conifer species heavily depend on fires, particularly crown fires, as its cones require high temperatures to open (Asselin et al. 2003; Kranz and Whitman 2019; Pelletier and de Lafontaine 2022; Smirnova et al. 2008). By contrast, the increase in individual fire sizes contributed to a steady decrease in abundance of fire‐sensitive conifer species such as balsam fir (from 1.5% down to 0%) (Figures 2H and 3E). Today, this species is limited to fire‐protected areas such as lakeshores and riverbanks (Ali et al. 2008; Jules et al. 2018; Sirois 1997).

### 3.2 Spring Drought Drives Fire Size

Downscaled simulations of VPD and ASW from the TraCE‐21ka (‐II) model yield additional understanding of Holocene hydrological dynamics. Over the last 8000 years, higher spring and summer VPD values consistently corresponded to lower simulated ASW (Figure 4; Spearman's rho = −0.95; *p* < 0.0001 and Spearman's rho = −0.97; *p* < 0.0001, respectively). Relationships between the FS index and seasonal means of these variables indicate that fire extents were predominantly driven by spring rather than summer conditions (Figure 4). Notably, fire size was positively correlated with spring VPD (Figure 4; Spearman's rho = 0.41; *p* < 0.0001) and negatively correlated with spring ASW (Figure 4; Spearman's rho = −0.47; *p* < 0.0001). By contrast, relationships between drying, biomass burned, and fire frequency followed expected patterns during the summer season, with increased VPD and reduced ASW associated with higher fire frequency and biomass burned (Figure 4).

A rapid increase in spring and summer VPD, and a concurrent decrease in ASW around 7.0 ky BP, marks a significant hydroclimatic shift. This period was also characterised by relatively low winter precipitation (November–March water equivalent < 200 mm; Figure S3), which may have limited soil moisture recharge and reduced ASW carryover from the previous year into spring, thereby priming fuels for early‐season burning. This shift coincides with the lingering effects of ice sheet retreat, which likely delayed the regional climate dynamics (Viau and Gajewski 2009). These conditions gradually moderated throughout the mid‐Holocene. Since *c*. 3.0 ky BP, spring VPD has increased from a median of 3.5 hPa (4.0–3.0 ky BP) to 4.2 hPa (0.2–0 ky BP), reaching 5.3 hPa during ad 1950–1990 (Figure 2B). These changes in atmospheric dryness enhanced soil evaporation, reducing spring ASW from 36 to 34 mm, a 6.4% drop in Relative ASW (RASW, from 92.8% to 86.4%; Figure 2D). The change in ASW is exacerbated in June (minus 2.3 mm in ASW, or 7.4% in RASW), with levels during ad 1950–1990 reaching values typical of July and August (29–30 mm; Figure S4). In contrast to spring trends, summer VPD decreased from 7.0 to 3.0 ky BP and ASW increased (Figure 2A,C). Since 3.0 ky BP, summer VPD stabilised, with only a slight increase (< 0.5 hPa on average) along with stable ASW trends up to the modern baseline (Figure 2A,C).

Climate proxy records provided complementary insights into climatic trends. Chironomid‐based reconstructions indicate that mean August air temperatures peaked between 8.0 and 7.0 ky BP, reaching *c*. 3°C above the modern baseline (ad 1950; Figure 2F). Such a warmer summer climate fostered the expansion of thermophilous species such as white pine, which was historically more abundant in the study area (Fréchette et al. 2018). A rapid summer cooling of *c*. 2.5°C occurred between 7.0 and 6.0 ky BP, followed by a slower decline of *c*. 1°C until 2.0 ky BP. Finally, a 1°C warming between 2.0 and 1.0 ky BP was followed by a gradual return to modern values. Pollen‐based temperature reconstructions corroborate the long‐term summer cooling between 8.0 and 2.0 ky BP (Figure 2F). The short warming episode recorded only by chironomids (2.0–1.0 ky BP) likely corresponds to the Roman Climatic Optimum (hereafter RCO; 1.9–1.6 ky BP) and/or the Medieval Warm Period (hereafter MWP; 1.1–0.7 ky BP) (Bajolle et al. 2018; Delwaide et al. 2021), which cannot be distinguished due to the temporal resolution of our records. These fluctuations are consistent with TraCE‐21 ka (‐II) simulations (Figure S5). Testate amoebae from peatlands indicate dry conditions from 4.0 and 2.5 ky BP, followed by wetter conditions thereafter (Figure 2E). Collectively, these data support a warm and dry Holocene Thermal Maximum (HTM; 8.0–4.0 ky BP) across the Northern Hemisphere. During this period, summers were warmer and drier compared to the modern baseline (Bajolle et al. 2018; Fréchette et al. 2018; Paillard et al. 2023; Van Bellen et al. 2013), albeit with sizable spatial and temporal variability (Cartapanis et al. 2022; Viau and Gajewski 2009). The HTM was followed by the Neoglacial cooling, marked by cooler and wetter conditions since 4.0 ky BP (Filion 1984; Viau and Gajewski 2009). Despite this general summer trend, our simulations (Figure 2A–D) suggest increasing spring dryness during the Neoglacial, contrasting with HTM conditions. This seasonal divergence reflects declining temperature seasonality in northern latitudes due to orbital forcing, with spring warming and summer cooling over the mid‐ to late Holocene (Figure S3).

## 4 Discussion

Our results highlight key changes in the eastern North American boreal forest over the past 8000 years, marked by increasing pyrogenicity driven by shifts in climate conditions and fire regimes. Before 4.0 ky BP, forest stands dominated by balsam fir, eastern white cedar and white pine (Figure 3) resembled modern mixedwood forests found hundreds of kilometres to the south. During the cooler Neoglacial period (since 4.0 ky BP), fires became larger but less frequent—a pattern that appears counterintuitive given the modern positive relationship between temperature and burned area (Gaboriau et al. 2022; Jain et al. 2022). Earlier research suggests that increased individual fire size since the Neoglacial period was primarily driven by changing spring climatic conditions, particularly through insolation‐driven warming early in the fire season (Ali et al. 2012; Remy et al. 2017). Our study enhances this understanding by integrating multiple paleoecological proxies (pollen, chironomids, testate amoebae) with simulated VPD and ASW data. This comprehensive approach reveals the complex mechanisms governing fire and vegetation dynamics in the northeastern boreal forest, particularly emphasising how the interplay between spring warming and dryness shapes these patterns. We specifically identify the critical role of changing seasonal moisture dynamics during the 4000–0 cal. BP period, especially the reduced amplitude of the difference between spring and summer conditions. This climatic shift fostered conditions conducive to larger, though less frequent, fire events. These fires typically occurred during periods of increasing spring VPD coupled with decreasing spring ASW (Figure 2). The altered fire regime triggered cascading effects on vegetation composition, notably promoting the expansion of highly flammable jack pine, which further reinforced fire‐prone conditions across the landscape. Our results therefore underscore the lingering impact of climate on vegetation–fire interactions, ultimately shaping the current dominance of black spruce and jack pine in eastern North American boreal forests—a legacy of the large individual fire events having occurred 4000–3000 years ago.

A decrease in black spruce pollen abundance is recorded since 2.0 ky BP, while the pollen percentage of jack pine remained high in the lake sediment archives, with a notable steep increase during the last 1000 years (Figure 3F,G). Simultaneously, an increase in individual fire size is recorded (Figure 3H). These data suggest that forests have become more pyrogenic, shaped by more severe and larger fires. Although climate influences jack pine distribution and growth (Girardin, Guo, et al. 2024), this species fundamentally depends on severe fire for its regeneration and long‐term persistence in the landscape. Its serotinous cones are obligate pyriscent, requiring exposure to temperatures > 50°C to open, which typically occurs during high‐intensity fires (Cameron 1953).

Therefore, climate alone—through changes in ASW or VPD—is unlikely to drive major shifts in jack pine abundance unless it also promotes severe stand‐replacing fires. The combination of prolonged spring dryness and stable summer moisture likely intensified severe fire activity thereby contributing to a compositional shift toward more fire‐adapted species, favouring jack pine over black spruce in the recent period. Spring fires (May–June) occurring under drier conditions are more effective at reducing the thickness of the soil organic layer due to underdeveloped herbaceous and shrub cover, which normally preserves soil moisture, hence limiting fire severity. Absence of a dense plant cover at the onset of the fire season leaves the organic layer more vulnerable to combustion (Terrier et al. 2014). In turn, high fire intensity and near‐complete consumption of the organic layer can shift competitive dynamics in favour of species like jack pine that regenerate better on exposed mineral soils (Baltzer et al. 2021). Residual soil organic layer thickness, crucial for the regeneration of small‐seeded boreal tree species, is an important factor determining conifer resilience (Augustin et al. 2022; Baltzer et al. 2021). Spring fires also burn more intensely than summer fires, affecting seed viability differently across species. Jack pine is particularly well‐adapted to intense fires, quickly establishing after disturbance, while black spruce cones require more time to release seeds (Greene et al. 2013). Beyond its fire‐related traits, jack pine is particularly sensitive to cold‐season frost events—an ecological response distinct from that of other boreal species (Girardin, Guo, et al. 2024). The increase in jack pine pollen abundance during the past millennia, marked by spring warming and stable summer moisture, likely reflects climate conditions that simultaneously favoured its post‐fire regeneration and growth.

**FIGURE 5:**
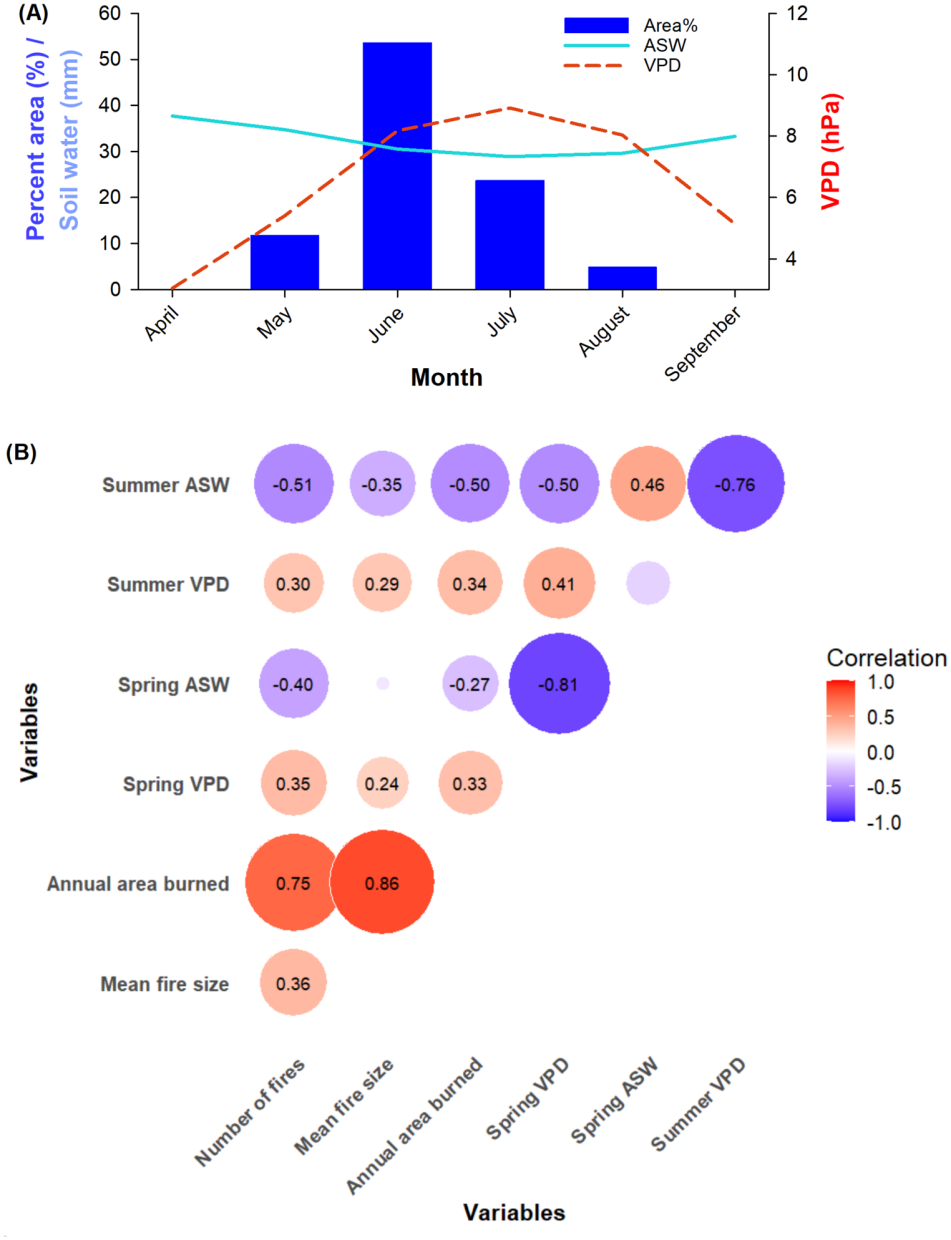
Relationships between modern seasonal dryness and fire regime components. (A) Summary of month‐to‐month variations in means of VPD and ASW, alongside the monthly percentages of the total burned area from 1950 to 2022. These percentages are calculated by summing the burned areas for each month across all years and comparing them to the total burned area over the period. (B) The Spearman correlation coefficient was utilised to quantify the strength of relationships, accounting for the persistence (memory) estimated for each time series. Results are visually represented using a heatmap diagram, illustrating by labels significant pairwise correlations among the variables at a 95% confidence level. Circles denote variable comparisons, with red indicating positive correlations and blue representing negative correlations. The size and colour gradient of the circles correspond to the magnitude of the correlation coefficients.

The climate simulation shows a steep increase in spring VPD during the last 250 years (Figure 2B), which suggests a recent amplification of dry environmental conditions during the spring fire season. Recent studies have underlined this climatic trend in boreal forests and identified VPD as a critical predictor of regional fire dynamics over extended periods (Clarke et al. 2022; Sedano and Randerson 2014; Wang et al. 2023). High spring VPD, combined with low spring ASW, significantly enhances fire activity—both in frequency and size—and extends these effects into summer (Figure 5 and Table S2). To illustrate the magnitude of these effects, a change in spring VPD from 4.9 to 6.3 hPa, and in spring ASW from 35.0 to 33.4 mm, is associated with more than a doubling in the annual incidence of fires exceeding 10 ha in size (from 23 to 63 fires per year; Table S3). Similarly, a change in summer VPD from 6.6 to 8.3 hPa, and in ASW from 31.2 to 30.1 mm, corresponds to a twofold increase in the number of fires (from 31 to 60 fires per year) and average fire sizes (923 to > 2372 ha), and a sizeable increase in the annual area burned (from 22,000 to > 143,000 ha annually).

## 5 Future Fire Activity in the Boreal Forest of Eastern North America: Lessons From the Past

The 8.0–7.0 ky BP period, marked by rapid warming (Figure 2F) and increasingly fire‐prone conditions, could share some similarities in rate and magnitude with anticipated future climate conditions. However, some differences must be considered. This period was characterised by a sharp increase in both spring and summer VPD, coupled with a sharp decline in summer ASW, leading to an abrupt rise in biomass burning (RegBB) (Figure 2G). During this period, fire regimes were predominantly summer‐driven, with peak fire occurrence during periods of maximum summer dryness (Figure S3). After 4.0 ky BP, spring droughts became increasingly pronounced during the fire season. This change has altered fire seasonality, with spring gradually presenting drought conditions like those in summer, particularly over the last 250 years (Figure 2B). Since that pivotal period, the conifer‐dominated boreal forests of northeastern North America have been shaped by infrequent but large and severe fires.

The extreme fire season of 2023 in Canada was driven by several factors, including persistent warm conditions from May to September, rapid snowmelt, and a dry spring. Fires ignited during the spring season (May–June) accounted for over two‐thirds of the total area burned (Jain et al. 2024). Our findings highlight a strong connection between spring environmental conditions and fire activity that has persisted for thousands of years and will likely continue to play a critical role in future fire dynamics. Notably, the VPD in eastern boreal North America is projected to increase at a rate of up to 0.05 hPa per year during the remainder of this century under a no‐policy baseline scenario (SSP5‐8.5), primarily driven by rising temperatures (Fang et al. 2022). Meanwhile, soil moisture is expected to decrease in the spring due to earlier snowmelt, with further reductions during the growing season because of higher evapotranspiration rates. Soil moisture during the entire fire season could thus be reduced by 20%–40% for the period 2070–2099 compared to the reference period (1971–2000) (Houle et al. 2012). These trends forecast extended and intensified fire seasons during the following decades (Augustin et al. 2022; Chaste et al. 2019).

Considering the predicted intensification of spring droughts and the cascading effect throughout the fire season, we posit that the boreal forest of eastern North America should continue to shift toward more pyrogenic vegetation, with consequences for wildlife habitats and biodiversity whereby fire‐sensitive species may struggle adjusting to changing landscapes (Morineau et al. 2023). In this scenario, fire‐prone conifer species, such as jack pine, will dominate the landscape. This perspective is inconsistent with some scenarios suggesting that the boreal coniferous forest will be invaded by thermophilous temperate forest species (Augustin et al. 2022; Chaste et al. 2019). Such projections may underestimate the role of fire as a recurrent disturbance agent and overestimate the capacity of broadleaf species to establish in boreal ecosystems. In particular, models that insufficiently account for the reinforcing feedback between fire and vegetation, the role of seasonal droughts in driving fire regimes, and the migration limitations of temperate species, whose northward dispersal may lag behind climate change velocity and disturbance‐driven openings (Talluto et al. 2017), may overpredict the pace and extent of biome shifts in this region. Bridging paleoecological evidence with dynamic modelling efforts will improve the realism of future projections by integrating both biotic and abiotic constraints on vegetation change, especially in fire‐prone systems such as the eastern North American boreal forest.

## Author Contributions

A.A.A., D.M.G., J.A.L., M.P.G. and C.C.R. designed the study and analysed data, created figures and drafted the initial version of the manuscript. M.G. and G.M. provided datasets for the study. All co‐authors have made significant contributions to the final manuscript.

## Peer Review

The peer review history for this article is available at https://www.webofscience.com/api/gateway/wos/peer‐review/10.1111/ele.70166.

## Supporting information


Data S1.

## Data Availability

The climate data used to support the findings of this study were obtained from https://climexp.knmi.nl/and https://trace‐21k.nelson.wisc.edu/Data.html. All relevant softwares and R‐functions that support the methods of this study are referred to in the “Material and methods” section (see package vignettes for details). The StandLEAP software used to generate past fluctuations in Vapour Pressure Deficit (VPD) and plant Available Soil Water (ASW) will be made available in Figshare: https://figshare.com/s/8c280d859278cfc8e810. Charcoal and pollen datasets were obtained from the Global Charcoal Database (https://paleofire.org/; accessed 2025‐05‐04) and the Neotoma Paleoecology Database (https://www.neotomadb.org/), respectively, or were provided directly by the original authors.
