# Supplementary material for: Drying Spring Accelerates Transitions Toward Pyrogenic Vegetation in Eastern Boreal North America
Source: Ecol Lett. 2025 Jun 26;28(6):e70166. doi: 10.1111/ele.70166 (PMC12202849; doi:10.1111/ele.70166)
Supplement: Supplementary file 1 — Data S1. [file ELE-28-0-s001.docx]

# **Supplemental information**

***SI text***

**RegBB**. The 17 individual CHAR series were pooled into a regional biomass burning history spanning 8,000 years (hereafter RegBB; unitless). Before regional pooling, individual CHAR series were homogenized to reduce the influence of sedimentation rate and potential taphonomic biases linked to the sequestration of charcoal in the lacustrine deposits (Ali *et al.* 2012; Marlon *et al.* 2008; Power *et al.* 2008). The procedure followed three different steps: (i) rescaling initial CHAR values using a min–max transformation, (ii) homogenizing the variance using Box–Cox transformation, and (iii) rescaling the values to z-scores. In the study zone, the RegBB approach has been validated against independent fire records, such as dendrochronological data (Ali *et al.* 2012) and from remote sensing data (Hennebelle *et al.* 2020), demonstrating its spatial and temporal reliability.

**RegFF**. In each CHAR series low-frequency variations (CHAR_background_) are separated from fire-related peaks (CHAR_peak_) using a locally-defined threshold and locally-weighted regression (Kelly *et al.* 2011). These peaks, which represent significant charcoal inputs from local fires, are further divided into noise (CHAR_noise_) and fire components (CHAR_fire_) using a Gaussian mixture model (Kelly *et al.* 2011). From fire event dates extracted from CHAR_fire_, we computed fire frequencies (hereafter FF; fire.year^1^), using a kernel density estimation procedure based on a 500-year smoothing bandwidth (Ali et al. 2012; Kelly et al. 2013). Then, the frequencies of fire events at each site were averaged into a regional fire frequency record (hereafter RegFF; fire.year^1^), by adjusting the values of FF to the changing number of lake samples through time (Ali *et al.* 2012).

**FS index** (RegBB/RegFF ratio). It is important to point out that this interpretation of the RegBB/RegFF ratio implicitly assumes that RegBB and RegFF represent fires that occurred over the same area and spatial scale, and to consider this index as a semi-quantitative proxy, pending field-study validation (Kelly *et al.* 2013). In our study zone, Hennebelle *et al.* (2020) provided empirical support for the FS index by linking lacustrine charcoal series to independently-derived data on area burned and fire severity (differenced Normalized Burn Ratio; dNBR). The annual charcoal particle deposition was correlated with the area burned and the two fire metrics (area burned and fire severity) were positively correlated.

**StandLEAP model**. This model relates absorbed photosynthetically active radiation to Gross Primary Production (GPP) using a radiation use efficiency (RUE) coefficient, adjusted by species-specific multipliers (*f*_1_…*f_n_*). Values below 1.0 indicate increasing environmental limitations (e.g., frost, VPD, ASW), whereas values above 1.0 (e.g., temperature) indicate conditions improving towards an optimum. In the StandLEAP model, the dynamic interaction between VPD, ASW, and transpiration plays a crucial role in water dynamics, influencing the overall moisture balance in the soil-tree-atmosphere continuum. Rising VPD amplifies the atmospheric evaporative demand, which in turn extracts more moisture from trees and soil. This increased moisture loss from trees occurs through transpiration, where water is drawn up from the roots and released as vapor through the leaves. Simultaneously, soil moisture decreases from higher evaporative demand. ASW is thus closely linked to both VPD and transpiration rates. The model runs on a monthly time step, with canopy light absorption and photosynthesis parameters derived from metadata that were generated using a more detailed multilayer, hourly time-step model of canopy photosynthesis and transpiration (Raulier *et al.* 2000). NPP is computed after partitioning respiration into maintenance (Rm) and growth (Rg: a fixed proportion of the difference between GPP and Rm) quantities and subtracting these from GPP. Rm is computed as a function of temperature and biomass. The model was validated against tree-ring data and eddy-covariance flux measurements across several sites in boreal Canada, and has shown good predictability (Girardin *et al.* 2011, 2016).

**References**

Ali, A.A., Blarquez, O., Girardin, M.P., Hely, C., Tinquaut, F., El Guellab, A., *et al.* (2012). Control of the multimillennial wildfire size in boreal North America by spring climatic conditions. *Proceedings of the National Academy of Sciences*, 109, 20966–20970.

Girardin, M.P., Bernier, P.Y. & Gauthier, S. (2011). Increasing potential NEP of eastern boreal North American forests constrained by decreasing wildfire activity. *Ecosphere*, 2, art25.

Girardin, M.P., Bouriaud, O., Hogg, E.H., Kurz, W., Zimmermann, N.E., Metsaranta, J.M., *et al.* (2016). No growth stimulation of Canada’s boreal forest under half-century of combined warming and CO2 fertilization. *Proceedings of the National Academy of Sciences*, 113, E8406–E8414.

Hennebelle, A., Aleman, J.C., Ali, A.A., Bergeron, Y., Carcaillet, C., Grondin, P., *et al.* (2020). The reconstruction of burned area and fire severity using charcoal from boreal lake sediments. *The Holocene*, 30, 1400–1409.

Kelly, R., Chipman, M.L., Higuera, P.E., Stefanova, I., Brubaker, L.B. & Hu, F.S. (2013). Recent burning of boreal forests exceeds fire regime limits of the past 10,000 years. *Proceedings of the National Academy of Sciences*, 110, 13055–13060.

Kelly, R.F., Higuera, P.E., Barrett, C.M. & Hu, F.S. (2011). A signal-to-noise index to quantify the potential for peak detection in sediment–charcoal records. *Quat. res.*, 75, 11–17.

Marlon, J.R., Bartlein, P.J., Carcaillet, C., Gavin, D.G., Harrison, S.P., Higuera, P.E., *et al.* (2008). Climate and human influences on global biomass burning over the past two millennia. *Nature Geosci*, 1, 697–702.

Power, M.J., Marlon, J., Ortiz, N., Bartlein, P.J., Harrison, S.P., Mayle, F.E., *et al.* (2008). Changes in fire regimes since the Last Glacial Maximum: an assessment based on a global synthesis and analysis of charcoal data. *Clim Dyn*, 30, 887–907.

Raulier, F., Bernier, P.Y. & Ung, C.-H. (2000). Modeling the influence of temperature on monthly gross primary productivity of sugar maple stands. *Tree Physiology*, 20, 333–345.

**Tables and Figures**

**Table S1.** Characteristics of the seventeen (17) lakes and three (3) fens used in the study.

| **Site** | **Latitude** | **Longitude** | **Year of sampling** | **Elevation (m asl)** | **Water depth (m)** | **Size (ha)** | **Length of organic sediment (cm)** | **Basal date**  **(cal. BP)** | **Proxies used in the study** | **Mean temporal resolution before statistical transformation (years)** | **Reference** |
| --- | --- | --- | --- | --- | --- | --- | --- | --- | --- | --- | --- |
| Lakes | | | | | | | | | | | |
| Aurélie | 50.418 | -74.230 | 2009 | 440 | 10 | 1 | 327 | 8279 | Chironomids | 52.6 | Bajolle 2018 |
|  |  |  |  |  |  |  |  |  | Pollen | 103.4 | Bajolle 2019 |
|  |  |  |  |  |  |  |  |  | Charcoal | 25 | El-Guellab 2015 |
| Pessière | 49.509 | -79.240 | 1997 | 280 | 16 | 4 | 584 | 7650 | Pollen | 111 | Carcaillet 2001 |
|  |  |  |  |  |  |  |  |  | Charcoal | 13 | Carcaillet 2001 |
| Schön | 50.595 | -77.568 | 2011 | 291 | 7 | 2.8 | 133 | 7297 | Pollen | 75.6 | Oris 2014 |
|  |  |  |  |  |  |  |  |  | Charcoal | 55 | Oris 2014 |
| Twin | 50.952 | -74.575 | 2009 | 376 | 5.7 | 2.9 | 184 | 8419 | Pollen | 90.4 | El-Guellab 2015 |
|  |  |  |  |  |  |  |  |  | Charcoal | 46 | El-Guellab 2015 |
| Marie Eve | 52.030 | -75.521 | 2011 | 296 | 8.7 | 16.5 | 290 | 6967 | Pollen | 68.0 | Oris 2014 |
|  |  |  |  |  |  |  |  |  | Charcoal | 24 | Oris 2014 |
| Nano | 53.024 | -77.364 | 2011 | 206 | 3.2 | 0.4 | 140 | 7450 | Pollen | 70.5 | Oris 2014 |
|  |  |  |  |  |  |  |  |  | Charcoal | 53 | Oris 2014 |
| Cèdre | 49.346 | -79.208 | 1997 | 315 | 15 | 2 | 600 | 7880 | Charcoal | 13 | Carcaillet 2006 |
| Garot | 51.100 | -77.554 | 2011 | 248 | 6.9 | 5.1 | 100 | 7379 | Charcoal | 74 | Oris 2014 |
| Geais | 49.892 | -78.655 | 2006 | 280 | 10.2 | 3.6 | 603 | 7939 | Charcoal | 13 | Ali 2009 |
| Lili | 49.174 | -79.374 | 2014 | 320 | 1.4 | 19 | 307 | 8449 | Charcoal | 28 | Remy 2018 |
| Loup | 53.055 | -77.401 | 2011 | 206 | 3 | 1.6 | 106 | 7191 | Charcoal | 68 | Oris 2014 |
| Loutre | 49.712 | -78.336 | 2006 | 274 | 10.6 | 2.1 | 227 | 7864 | Charcoal | 35 | Ali 2009 |
| Nans | 50.368 | -74.303 | 2009 | 431 | 5 | 4.5 | 230 | 8491 | Charcoal | 37 | El-Guellab 2015 |
| Profond | 49.861 | -78.613 | 2006 | 270 | >20 | 0.6 | 223 | 4025 | Charcoal | 18 | Ali 2009 |
| Raynald | 49.809 | -78.536 | 2006 | 250 | 10.3 | 1.5 | 472 | 7060 | Charcoal | 15 | Ali 2009 |
| Richard | 50.645 | -74.679 | 2009 | 432 | 5.3 | 1.2 | 165 | 8265 | Charcoal | 50 | El-Guellab 2015 |
| Trèfle | 51.965 | -76.081 | 2011 | 270 | 5.4 | 6.8 | 150 | 7139 | Charcoal | 48 | Oris 2014 |
|  | Fens | | | | | | | | | | |
| Aero1 | 54.101 | -72.516 | 2005 | 430 | / | 3.0 | 100 | 5000 | Testate amoebae | 105.25 | Van Bellen 2013 |
| Aero5 | 54.101 | -72.516 | 2005 | 430 | / | 3.0 | 100 | 4469 | Testate amoebae | 161.4 | Van Bellen 2013 |
| Abeille5 | 54.114 | -72.500 | 2010 | 440 | / | 3.5 | 100 | 3623 | Testate amoebae | 146.7 | Van Bellen 2013 |
| Ours1 | 54.049 | -72.457 | 2005 | 460 | / | 1.6 | 100 | 5522 | Testate amoebae | 206.3 | Van Bellen 2013 |
| Ours4 | 54.049 | -72.457 | 2005 | 460 | / | 1.6 | 100 | 4918 | Testate amoebae | 183.6 | Van Bellen 2013 |

**Table S2.** Spearman correlations for the relationships among fire regime parameters and ASW and VPD seasonal variables. Statistical significance was assessed considering the autocorrelation for each time series. The null hypothesis of no significant correlation is rejected at the 5% level when the 95% bootstrap confidence interval (CI) excludes zero. Significant correlations are indicated by an asterisk.


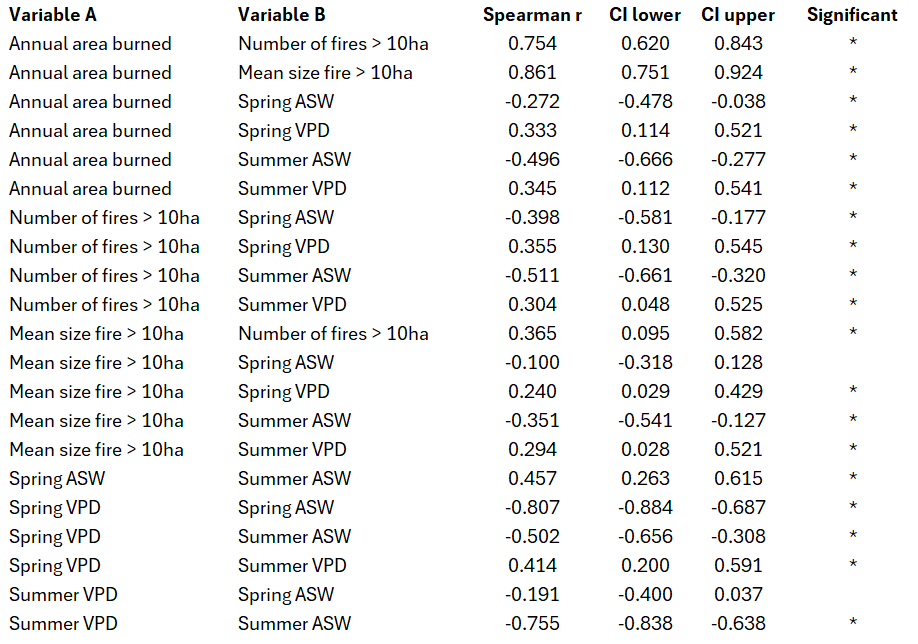


**Table S3.** Differences in the medians of annual area burned, mean fire size and number of fires during the ten years of lowest and highest VPD and ASW conditions, during the period 1950 to 2022. Statistical significance of Student *t* at the 5% level for differences in medians was assessed using accelerated bootstrap confidence intervals (BCa). The null hypothesis of no significant difference in medians is rejected at the 5% level when BCa upper and lower bounds exclude zero. Significant differences appear in bold.


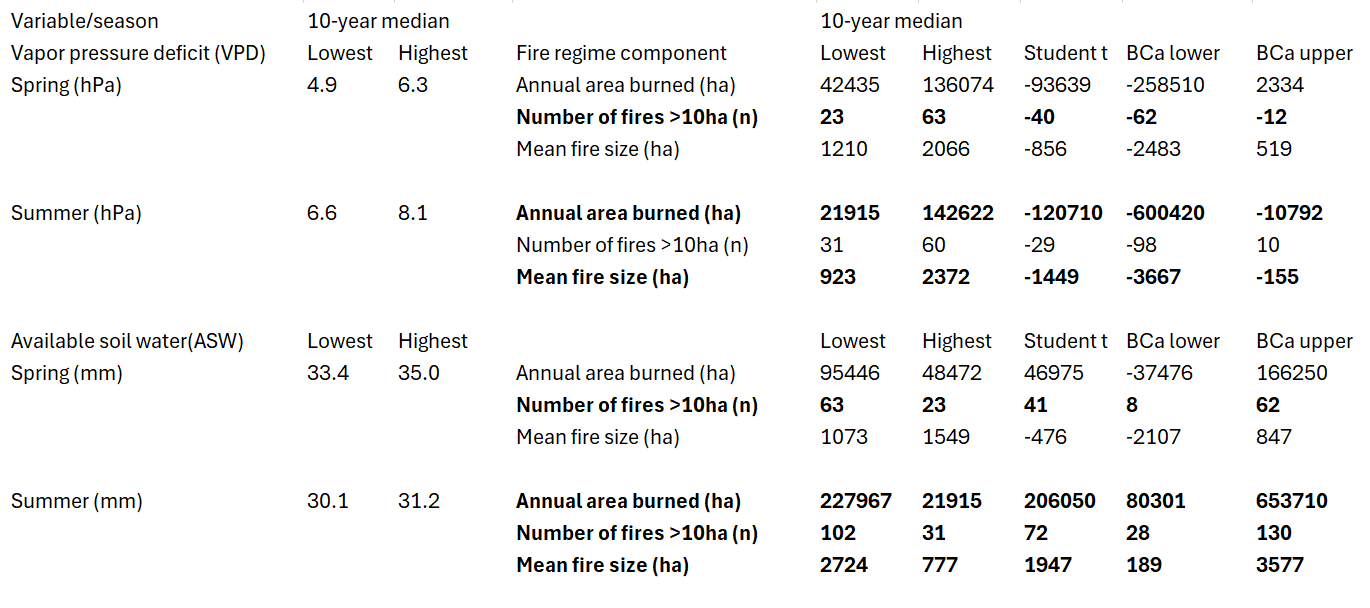


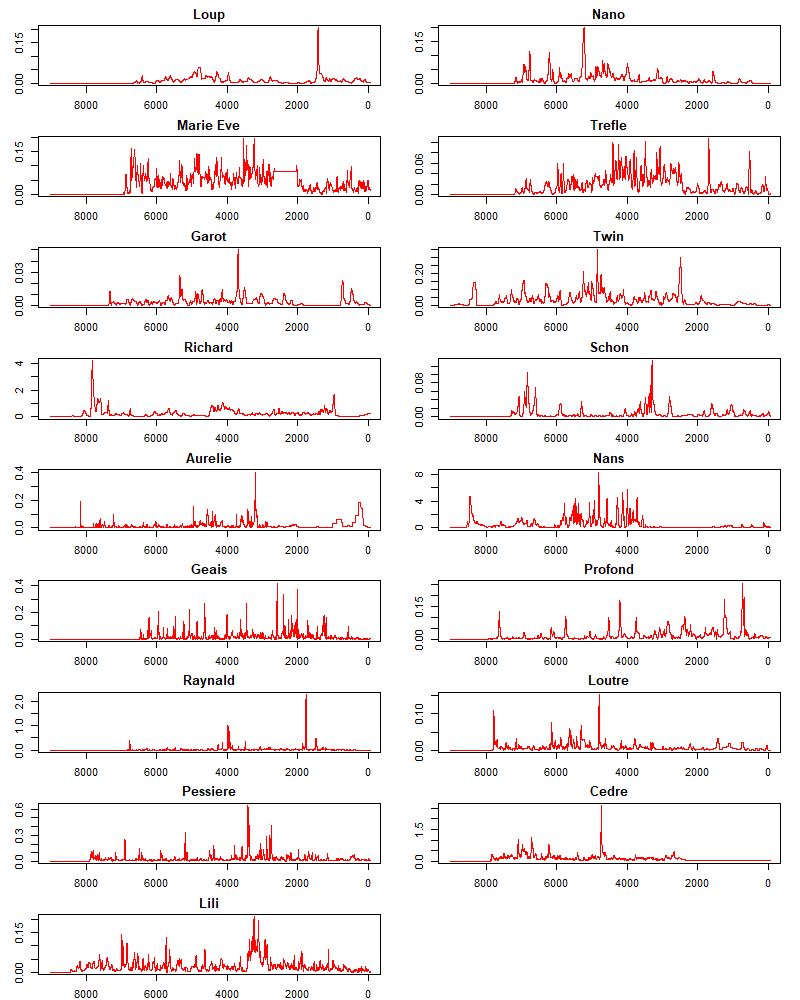


**Figure S1.** Individual charcoal accumulation rates (mm^2^.cm^2^.year) obtained from CHARanalysis and for each of the 17 study lakes. To remove bias induced by variations in sedimentation rate at the site level, we interpolated individual CHAR series using a constant time resolution corresponding to the median sample resolution of each lake.


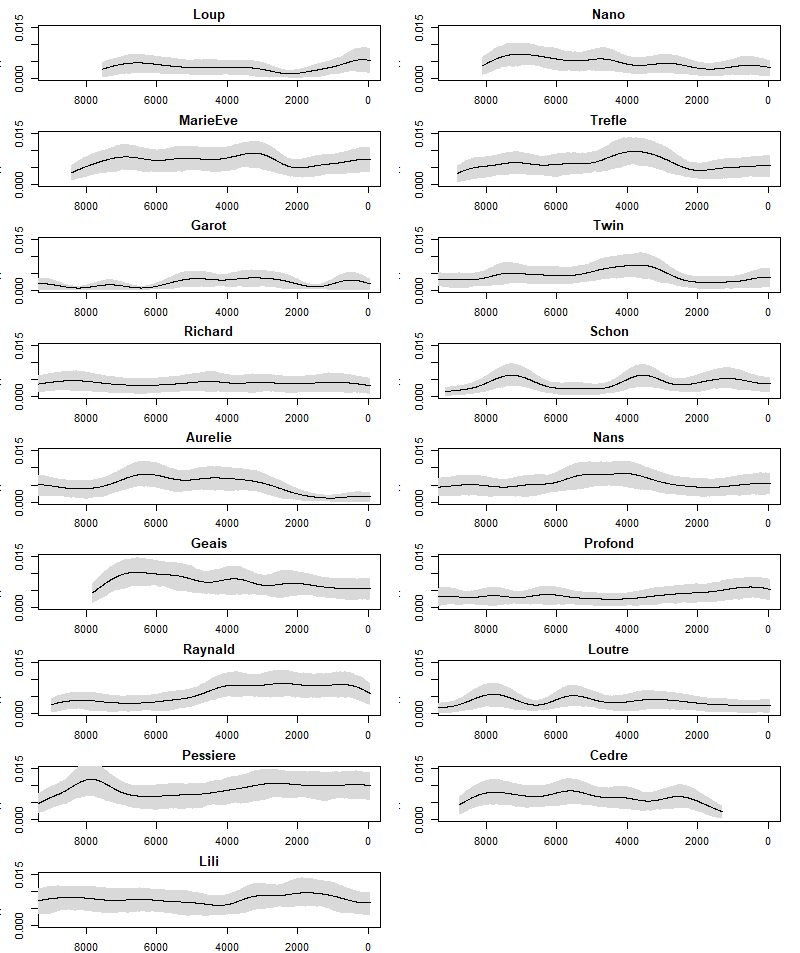


**Figure S2.** Annual fire frequencies obtained from CHARanalysis for each of the 17 study lakes.


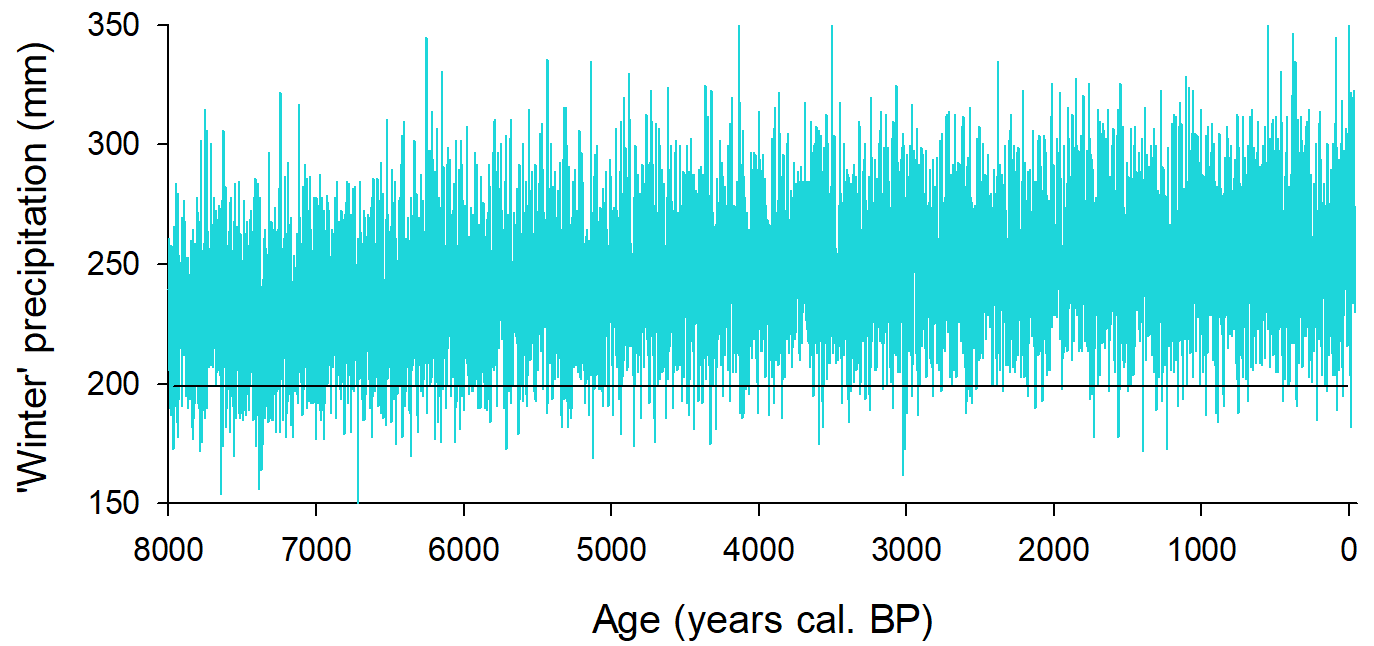


**Figure S3.** Total cold-season precipitation (mm water equivalent; November–March) reconstructed from the TraCE-21ka-II transient climate simulation over the period from 8,000 to 0 cal. years BP. The 200 mm threshold (horizontal dashed line) is used as a benchmark to identify years of low winter precipitation that may have contributed to limited soil moisture recharge and increased susceptibility to spring drought. This threshold is grounded in empirical observations and fire science literature, which indicate that overwinter recharge of soil moisture and forest floor fuels in boreal systems typically require a minimum of ~200 mm of cold-season precipitation. When this threshold is not met, insufficient snow accumulation and melt can result in low available soil moisture (ASW) in early spring, a key factor in drying forest fuels and increasing fire potential. Years below this 200 mm threshold helps isolate intervals that may have been particularly prone to spring dryness and elevated fire risk (Turner and Lawson 1978).


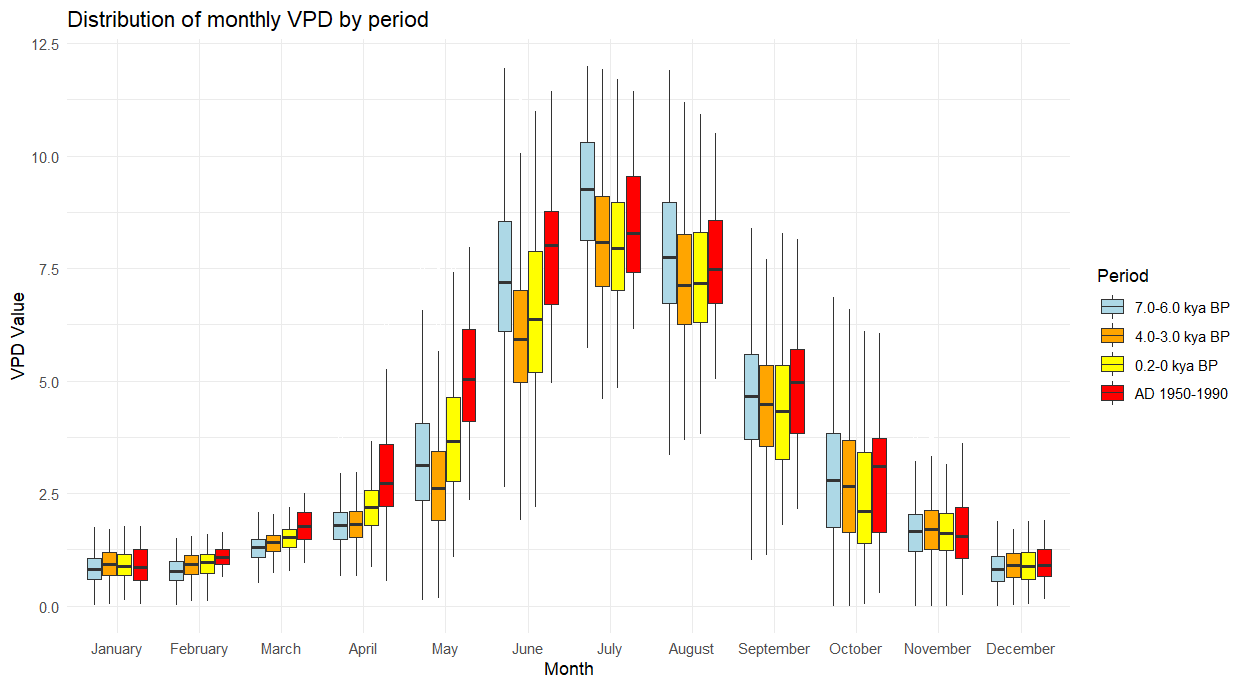

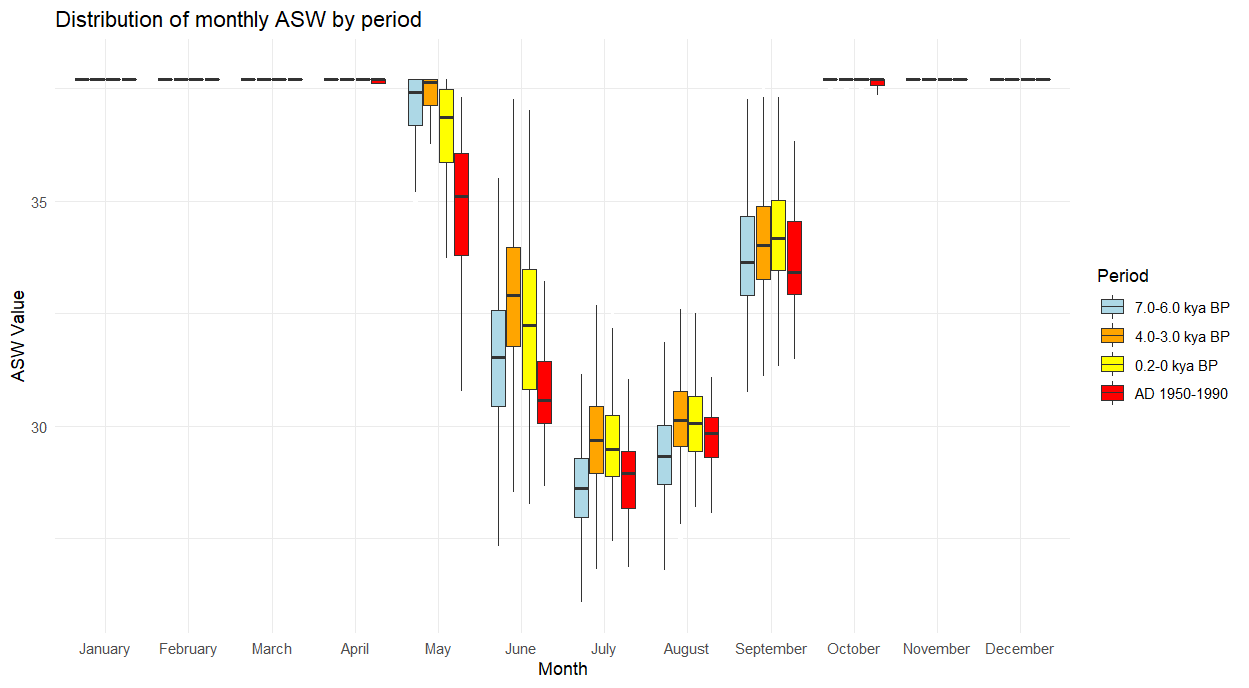


**Figure S4.** Seasonal variation in monthly available soil water (ASW) and vapor pressure deficit (VPD) across historical periods. Median and interquartile range (IQR) are displayed in boxplots. The whiskers extend from the edges of the box to show the range of typical ASW values within 1.5 times the IQR from the quartiles. Sample sizes for the three periods are respectively *n* = 1001, 200 and 41 years.


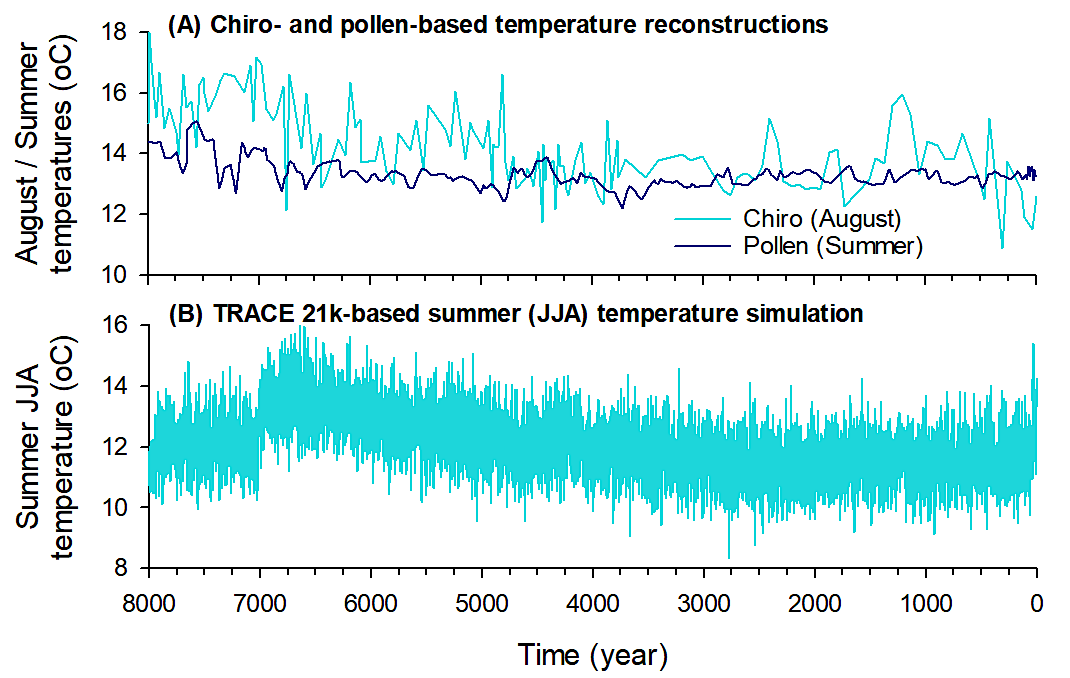


**Figure S5**. Comparison of proxy-inferred and simulated temperatures from 8,000 cal. BP to present (AD 1990). (A) Chironomid-inferred August and pollen-inferred summer temperature reconstructions. (B) Simulated summer temperature from TRACE-21k (-II) climate model output averaged across the whole study domain.
